# Supplementary material for: Urinary proteome profiling for stratifying patients with familial Parkinson’s disease
Source: EMBO Mol Med. 2021 Jan 22;13(3):e13257. doi: 10.15252/emmm.202013257 (PMC7933820; doi:10.15252/emmm.202013257)
Supplement: Supplementary file 1 — Appendix [file EMMM-13-e13257-s001.docx]

**Appendix**

**Urinary proteome profiling for stratifying patients with familial Parkinson’s disease**

Sebastian Virreira Winter^1,a,b^, Ozge Karayel^1,a^, Maximilian T Strauss^1,b^, Shalini Padmanabhan^2^, Matthew Surface^3^, Kalpana Merchant^4^, Roy N. Alcalay^3^, Matthias Mann^1,5,c^

^1^ Department of Proteomics and Signal Transduction, Max Planck Institute of Biochemistry, Martinsried, Germany

^2^ The Michael J. Fox Foundation for Parkinson’s Research, NY, USA

^3^ Department of Neurology, Columbia University, NY, USA

^4^ Northwestern University Feinberg School of Medicine, IL, USA

^5^ Novo Nordisk Foundation Center for Protein Research, Faculty of Health Sciences, University of Copenhagen, Copenhagen, Denmark

^a^ These authors contributed equally

^b^ Current address: OmicEra Diagnostics GmbH, Behringstr. 6, 82152 Planegg, Germany

^c^ Corresponding author

Correspondence: [mmann@biochem.mpg.de](mailto:mmann@biochem.mpg.de)

**Table of Content**

Appendix Figure S1. Clinical information and library depth page 2

Appendix Figure S2. Assessment of the quantification precision page 3

Appendix Figure S3. Quality assessment of urine samples page 5

Appendix Figure S4. Correlation analysis page 6

Appendix Figure S5. Decision-tree-based feature selection for machine learning page 7

**
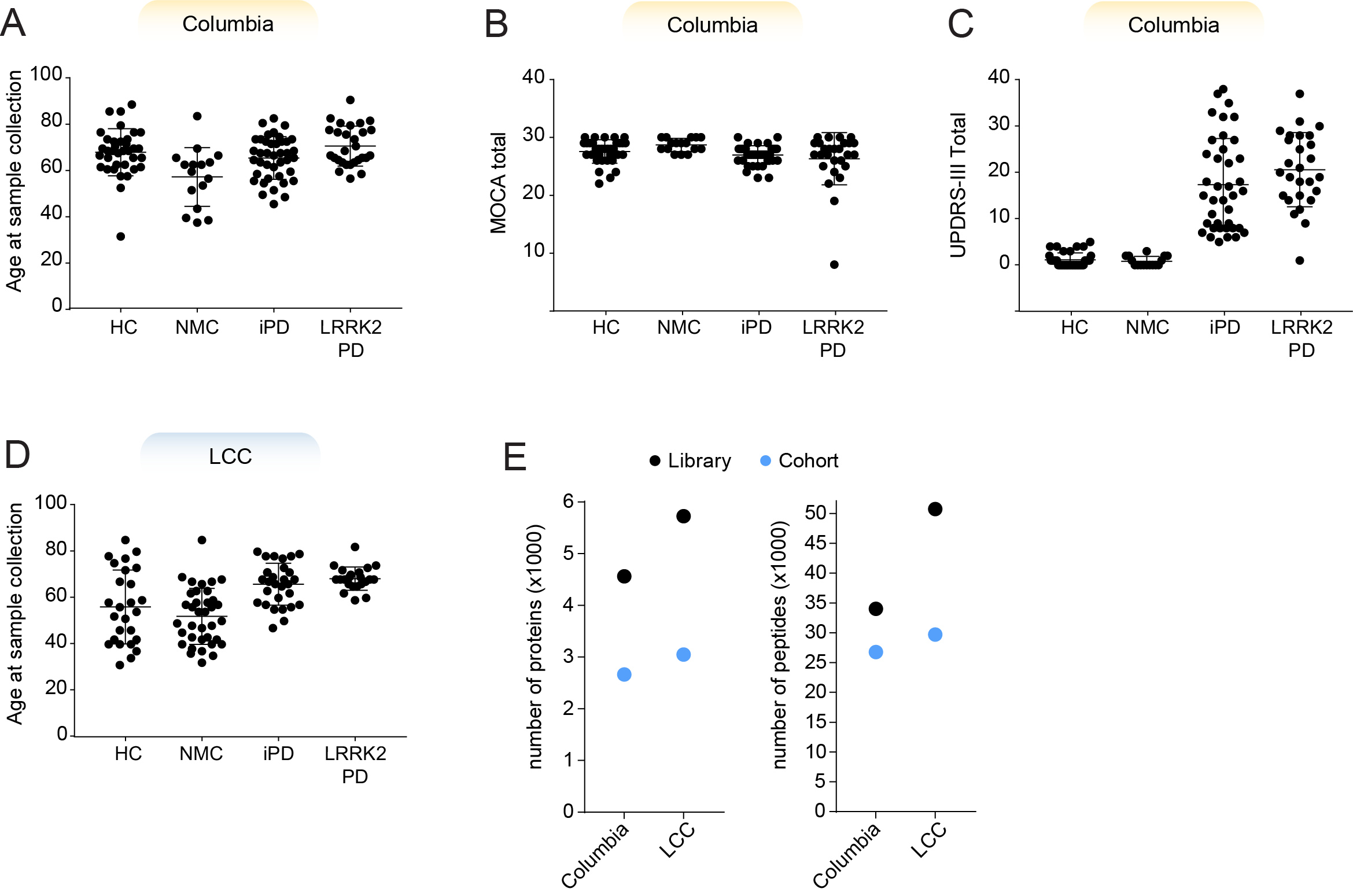
**

**Appendix Figure S1. Clinical information and library depth**

**A**) Age of subjects at the time of sample collection for all participants of the Columbia cohort. Bars represent mean and standard deviation in all panels.

**B-C**) Clinical scores from MoCA (B) and UPDRS-III (C) for all individuals of the Columbia cohort.

**D**) Age of subjects at the time of sample collection for all participants of the LCC cohort.

**E**) Number of proteins and peptides identified in each cohort-specific hybrid library.


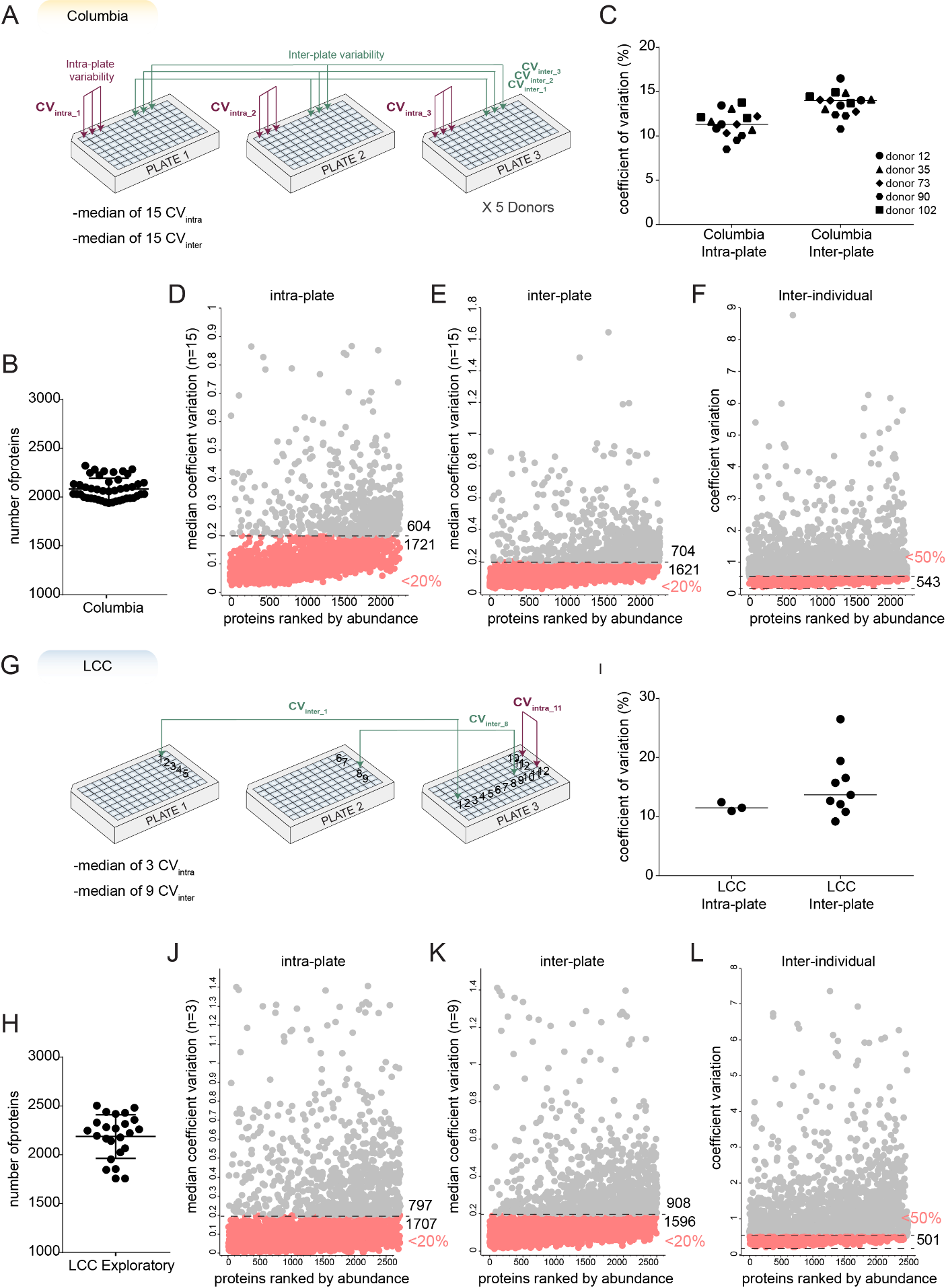


**Appendix Figure S2. Assessment of the quantification precision**

**A**) Graphical overview of the experiment to determine coefficients of variation (CVs) for the quantification of proteins in the Columbia cohort.

**B**) Number of proteins quantified in each sample for the CV determination experiment of the Columbia cohort.

**C**) Median intra- and inter-plate CV values for the Columbia cohort.

**D-E**) Median intra-plate (D) and inter-plate (E) CVs for each protein quantified in the Columbia cohort. Proteins were ranked according to their abundance and proteins with a median CV below 20% are highlighted in pink. Numbers of proteins above and below this CV threshold are given.

**F**) Inter-individual CVs were calculated from the analysis of every individual in the Columbia cohort. Proteins with a CV below 50% are highlighted in pink.

**G**) Graphical overview of the experiment to determine coefficients of variation (CVs) for the quantification of proteins in the LCC cohort.

**H**) Number of proteins quantified in each sample for the CV determination experiment of the LCC cohort.

**I**) Median intra- and inter-plate CV values for the LCC cohort.

**J-K**) Median intra-plate (D) and inter-plate (E) CVs for each protein quantified in the LCC cohort. Proteins were ranked according to their abundance and proteins with a median CV below 20% are highlighted in pink. Numbers of proteins above and below this CV threshold are given.

**L**) Inter-individual CVs were calculated from the analysis of every individual in the LCC cohort. Proteins with a CV below 50% are highlighted in pink.


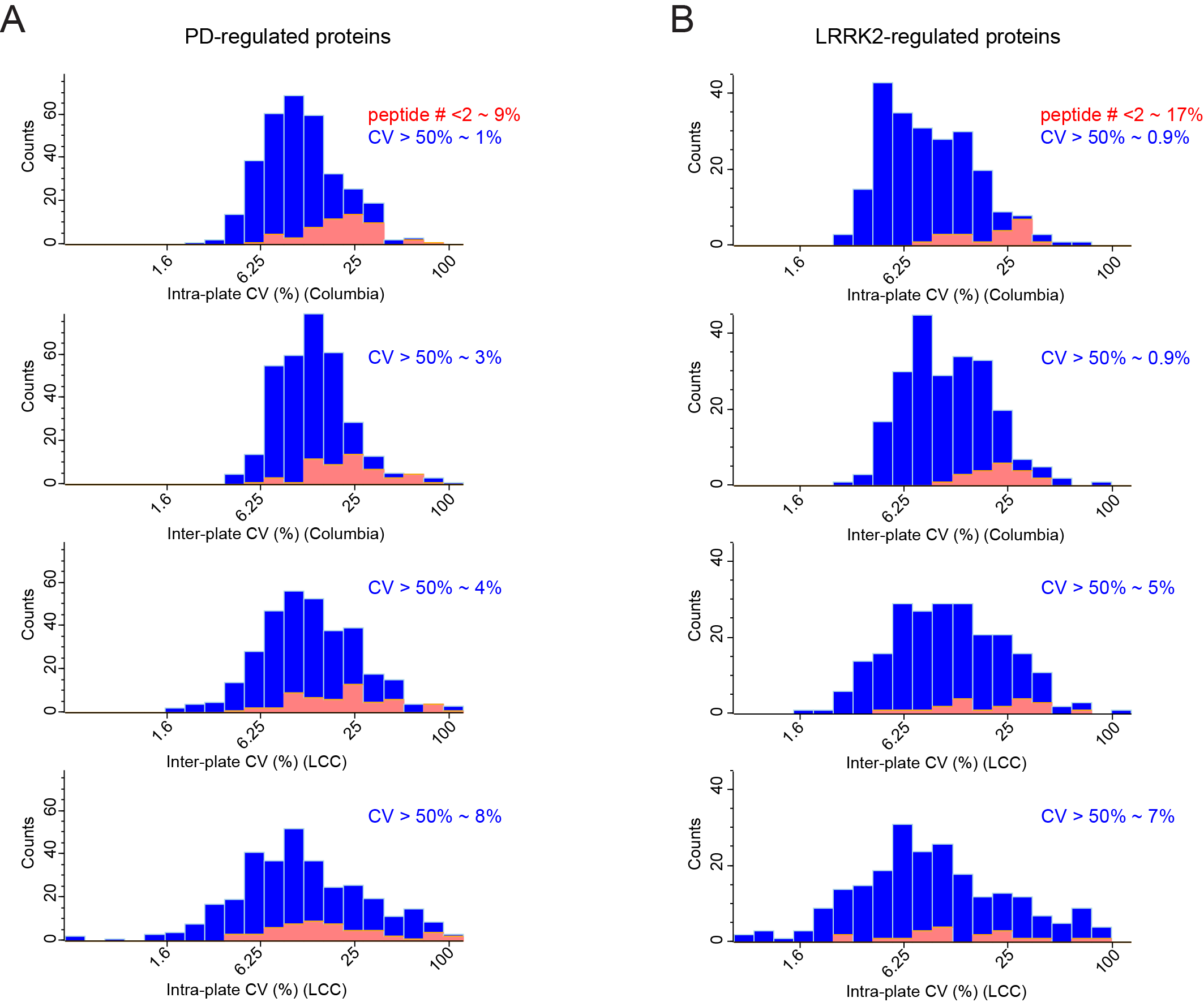


**Appendix Figure S3. Quality assessment of urine samples**

**A-B**) The quantification precision shown as intra- or inter-plate CVs for the Columbia and LCC cohorts for (A) PD-regulated proteins and (B) *LRRK2*-regulated proteins.


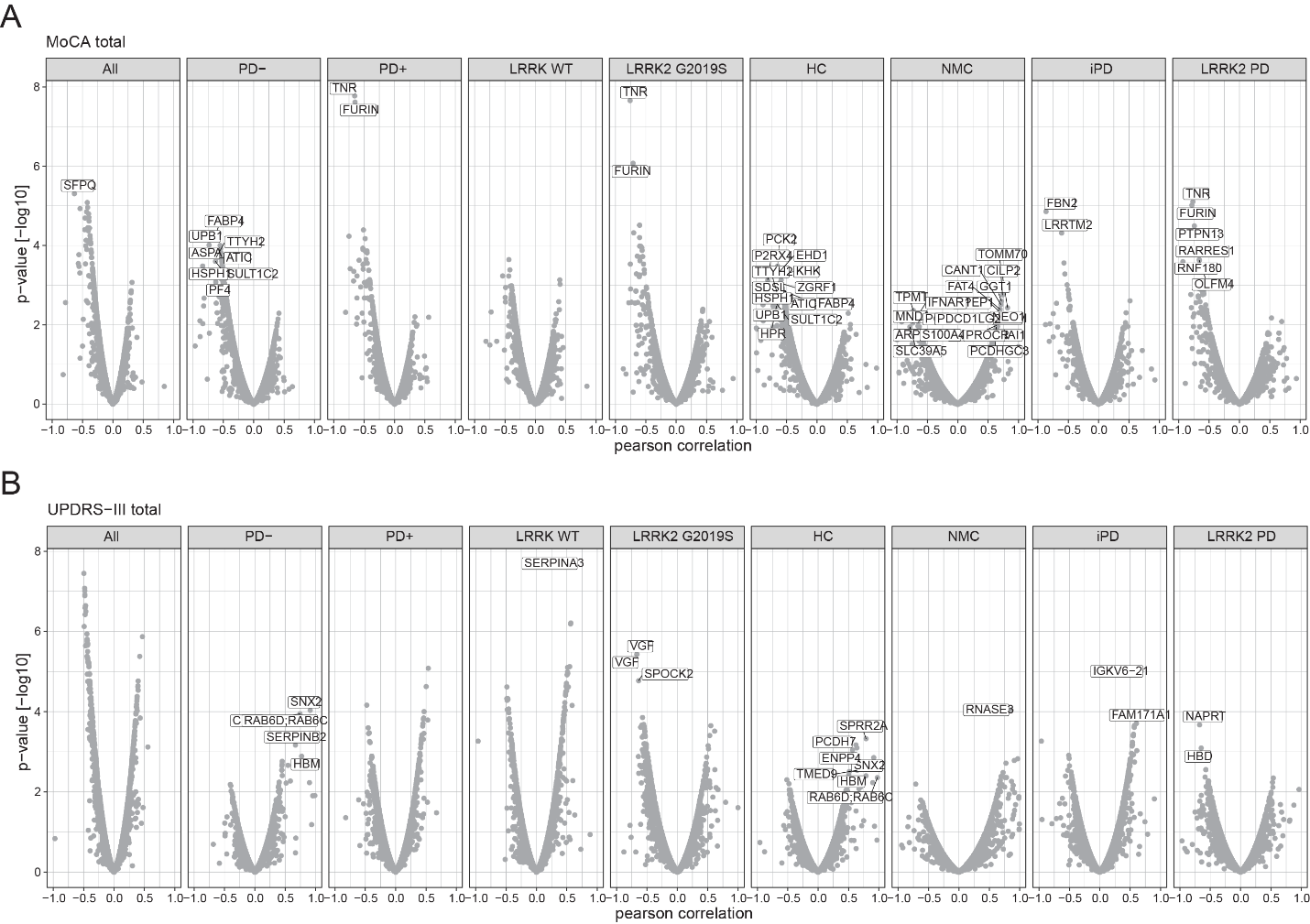


**Appendix Figure S4. Correlation analysis**

**A**) Pearson correlation scores and associated p-values [-log10] of all protein intensities with the MoCA total score. The subset of individuals included in the analyses is shown on top.

**B**) Pearson correlation scores and associated p-values [-log10] of all protein intensities with the UPDRS-III score. The subset of individuals included in the analyses is shown on top.

**
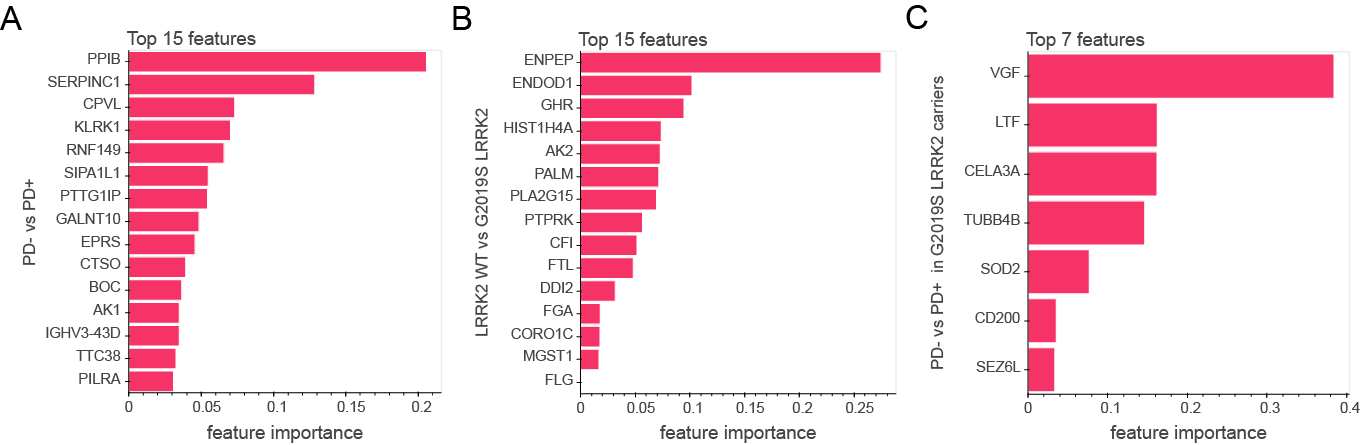
**

**Appendix Figure S5. Decision-tree-based feature selection for machine learning**

**A**) Top15 most important features according to a decision tree-based feature selection to classify PD+ vs. PD- individuals.

**B**) Top15 most important features according to a decision tree-based feature selection to classify *LRRK2* G2019S vs. *LRRK2* WT carriers.

**C**) Top7 most important features according to a decision tree-based feature selection to classify PD+ vs. PD- in *LRRK2* G2019S carriers.
